# Stochasticity of replication forks’ speeds plays a key role in the dynamics of DNA replication

**DOI:** 10.1371/journal.pcbi.1007519

**Published:** 2019-12-23

**Authors:** Razie Yousefi, Maga Rowicka

**Affiliations:** 1 Department of Biochemistry and Molecular Biology, University of Texas Medical Branch at Galveston, Galveston, Texas, United States of America; 2 Institute of Translational Sciences, University of Texas Medical Branch at Galveston, Galveston, Texas, United States of America; University of California Irvine, UNITED STATES

## Abstract

Eukaryotic DNA replication is elaborately orchestrated to duplicate the genome timely and faithfully. Replication initiates at multiple origins from which replication forks emanate and travel bi-directionally. The complex spatio-temporal regulation of DNA replication remains incompletely understood. To study it, computational models of DNA replication have been developed in *S. cerevisiae*. However, in spite of the experimental evidence of forks’ speed stochasticity, all models assumed that forks’ speeds are the same. Here, we present the first model of DNA replication assuming that speeds vary stochastically between forks. Utilizing data from both wild-type and hydroxyurea-treated yeast cells, we show that our model is more accurate than models assuming constant forks’ speed and reconstructs dynamics of DNA replication faithfully starting both from population-wide data and data reflecting fork movement in individual cells. Completion of replication in a timely manner is a challenge due to its stochasticity; we propose an empirically derived modification to replication speed based on the distance to the approaching fork, which promotes timely completion of replication. In summary, our work discovers a key role that stochasticity of the forks’ speed plays in the dynamics of DNA replication. We show that without including stochasticity of forks’ speed it is not possible to accurately reconstruct movement of individual replication forks, measured by DNA combing.

## Author summary

DNA replication in eukaryotes starts from multiple sites termed replication origins. Replication timing at individual sites is stochastic, but reproducible population-wide. Complex and not yet completely understood mechanisms ensure that genome is replicated exactly once and that replication is finished in time. This complex spatio-temporal organization of DNA replication makes computational modeling a useful tool to study replication mechanisms. For simplicity, all previous models assumed constant replication forks’ speed. Here, we show that such models are incapable of accurately reconstructing distances travelled by individual replication forks. Therefore, we propose a model assuming that replication speed varies stochastically between forks. We show that such model reproduces faithfully distances travelled by individual replication forks. Moreover, our model is simpler than previous model and thus avoids over-learning (fitting noise). We also discover how replication speed may be attuned to timely complete replication. We propose that forks’ speed increases with diminishing distance to the approaching fork, which we show promotes timely completion of replication. Such speed up can be e.g. explained by a synergy effect of chromatin unwinding by both forks. Our model can be used to simulate phenomena beyond replication, e.g. DNA double-strand breaks resulting from broken replication forks.

## Introduction

DNA replication in eukaryotic cells is highly regulated to ensure that the whole genome is duplicated correctly and completely before cell division [1]. Replication initiates at specific sites, termed origins of replication. Origins are prepared to be activated (i.e. fired) with the assembly of a pre-replication complex, through a process termed licensing, during the G1 phase [2]. Replication origins are licensed in excess and during the subsequent S phase a subset of origins initiate replication. Two forks emanate and elongate bi-directionally from each active origin, the rest of the licensed origins are passively replicated by the forks emerging from the neighbor origins [3, 4]. In the budding yeast *Saccharomyces cerevisiae*, DNA replication initiates from ∼400 origins with known genomic coordinates [5]. Origin activation is stochastic in individual cells [6, 7], but chronological order of origin activation is reproducible population-wide. This flexibility in origin activation is essential in response to DNA damage and adaption of replication to gene expression [8, 9]. Upon origin activation, replication forks are formed and progress along the chromosome until they meet another fork moving in the opposite direction. High-throughput experimental data, which have been used to study the dynamics of DNA replication, allow the measurement of average replication time and average forks’ speed, but mask variations in these parameters between forks [10]. Distances travelled by individual replication forks *in vivo* can be visualized and measured using DNA combing. However, DNA combing does not provide the genomic coordinates, and complexity of spatio-temporal regulation of replication makes interpretation of these data difficult. As a result, computational models are necessary to analyze the mechanism of DNA replication and understand how regulation of origin activation and fork elongation impact its dynamics.

Substantial stochasticity of replication forks’ speeds has been observed in *in vitro* biophysical studies of individual forks [11] and in DNA combing and 2D gel analysis in *S. cerevisiae* [11–16]. Nevertheless, previous models assumed that forks’ speed was not stochastic (i.e. did not vary between forks) [7, 17–30]. Moreover, previous models used only population-wide data and typically employed origin-to-origin comparison for validation and parameter selection [22–24, 30]. Such an approach can elucidate information about origin average firing time and efficiency (i.e. percentage of cells in which origin is fired), but it cannot distinguish between variability in the forks’ speeds and the stochasticity of origin firing time.

Here, we present Repli-Sim, a probabilistic numerical model for DNA replication, which simulates DNA replication in *S. cerevisiae* genome-wide assuming stochastic replication forks’ speeds. Repli-Sim includes local parameters specific to each origin inferred from experimental data and global parameters assigned to origins using a Monte Carlo method and optimized through a genetic algorithm. We used both data on distances travelled by individual replication forks (DNA combing) and cell population-wide measurements (DNA copy number data) to validate our model. We show that stochasticity in the forks’ speeds is key to reconstructing dynamics of DNA replication in single cells, as measured by DNA combing. We also show that constant forks’ speed models, such as previously used, are incapable of accurately reconstructing distribution of distances traveled by individual replication forks, as measured by DNA combing. We also report the observation, based on three independent datasets, that an individual fork speed may depend on the distance to the approaching fork. We show that such modification of the fork speed promotes timely completion of the replication, which is a challenge due to its stochastic nature.

## Results

We will use both a single origin of replication and the whole genome to show how the variance of forks’ progression rate impacts the distribution of distances travelled by individual forks, i.e. the so called DNA tracks. For single origin of replication analysis, we will illustrate a significant increase in the difference between variable and constant forks’ speeds at later times during S phase, representing a more dominant effect of forks’ progression rate variability at longer times, while the average length of the DNA track remains comparable in all models. For genome wide analyses, Repli-Sim is utilized to derive the DNA tracks for both untreated and hydroxyurea-treated cells and it is shown how taking into account variability in replication forks’ speeds impacts the dynamics of replication and distribution of DNA tracks.

### Repli-Sim

**Fig 1:**
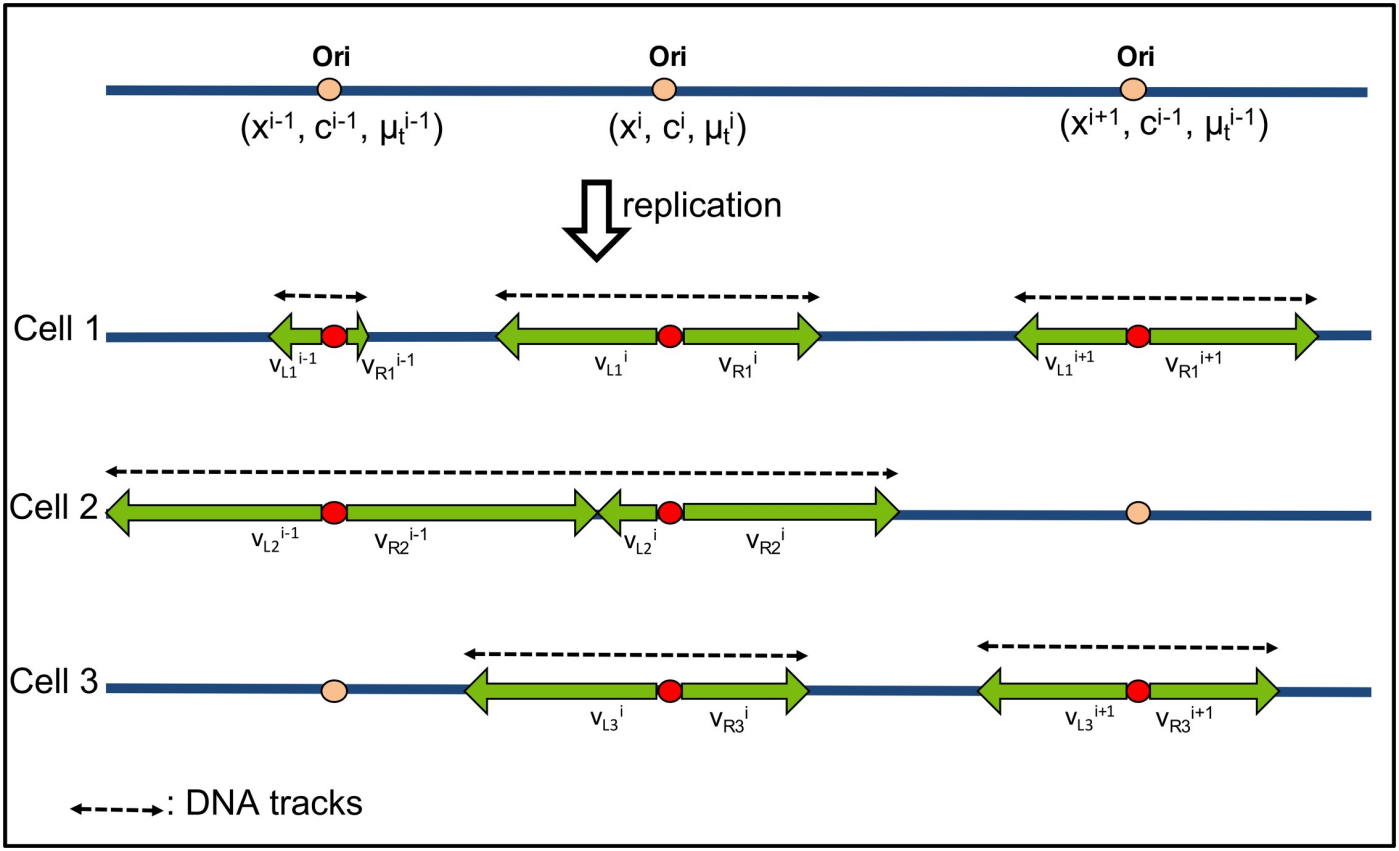
A schematic of the mechanism of DNA replication encoded in Repli-Sim. Repli-Sim includes local origin parameters (position *x*^*i*^, competence *c*^*i*^, and mean firing time $\mu_{t}^{i}$) and global parameters (firing time variance *σ*_*t*_, mean forks’ speed *μ*_*v*_ and its variance *σ*_*v*_). When an origin of replication activates, two forks are formed and elongate bidirectionally until they meet an approaching fork. The speed of an individual fork is constant, but vary between forks, even if they emanate from the same origin. The continuous length of the replicated DNA (Δ*x*, DNA tracks) are shown with the dashed lines.

Repli-Sim is a probabilistic numerical model designed to study the dynamics of DNA replication. Origins of replications are licensed (i.e. prepared) to be activated during G1 phase of cell cycle, the frequency with which a given origin is licensed is called its competence, *c*^*i*^. During S-phase, licensed origins of replication are either activated or they are passively replicated by other forks. DNA tracks (continuous distances covered by replication forks, Fig 1) are formed and elongated throughout the genome until the whole DNA is replicated. In Repli-Sim (Methods), coordinates *x* of replication origins are derived from experimental data and filtered using a database of replication origins, OriDB [5]. As shown in Fig 1, two forks are formed and elongate bidirectionally across the genome to form DNA tracks (Δ*x*). For each origin *i* in a cell population, at time *t*_*exp*_ (measured from the beginning of DNA replication), we derive the distribution of Δ*x* based on two assumptions. First, the firing time of the origin, $t_{0}^{i}$, is derived from a normal distribution with a mean firing time $\mu_{t}^{i}$ (specific to that origin and derived from experimental data), and with global standard deviation *σ*_*t*_:

$$\begin{array}{c} t_{0}^{i}\leftarrow N\left(\mu_{t}^{i}|\sigma_{t}^{2}\right). \end{array}$$ (1)

Second, individual forks are assigned with different speeds, *v*^*i*^, derived from the same probability distribution with a mean speed, *μ*_*v*_ and standard deviation *σ*_*v*_:

$$\begin{array}{c} v^{i}\leftarrow N\left(\mu_{v}|\sigma_{v}^{2}\right). \end{array}$$ (2)

A probability of origin licensing *c*^*i*^ (competence, i.e. a priori probability of origin activation) is assigned to each individual origin as a random number between the experimentally measured frequency of that origin activation and 1. This probability is used to determine which origins are activated and a Monte-Carlo method is used to generate activation time $t_{0}^{i}$ for an origin *i* from a Gaussian probability distribution with an experimentally estimated mean activation time $\mu_{t}^{i}$ specific to that origin (Methods), and a global standard deviation *σ*_*t*_, same for each origin. Origins passively replicated (i.e. replicated by a fork emanating from another origin) are identified and removed from calculations. Individual forks progress with different speeds, constant for each fork, generated using a Monte-Carlo method from a Gaussian probability distribution with a global average speed *μ*_*v*_ and standard deviation *σ*_*v*_. Forks stop when they encounter a fork traveling from another direction.

### Impact of stochasticity of forks’ speeds on the dynamics of DNA replication

**Fig 2:**
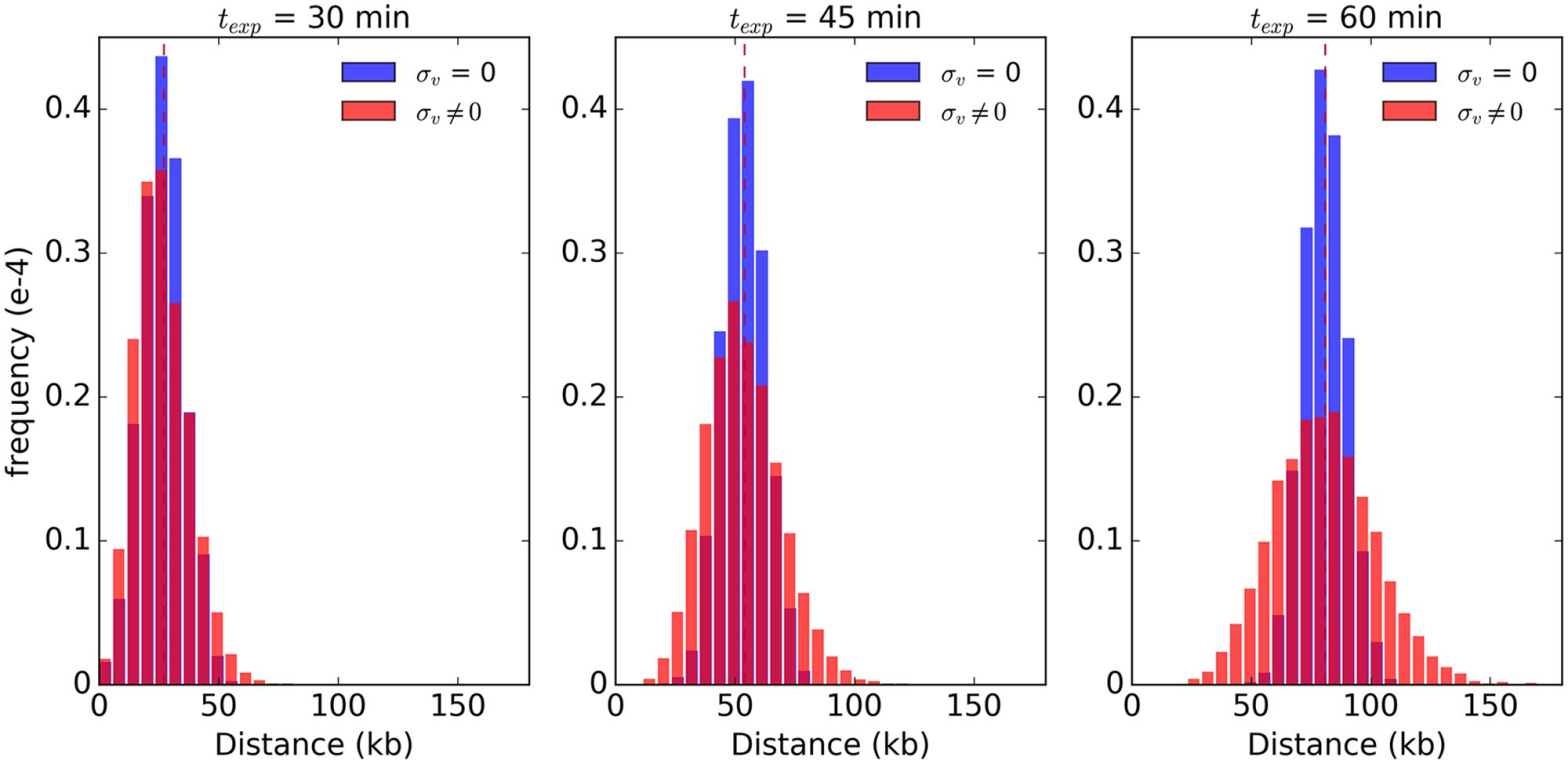
The impact of stochasticity of replication forks’ speeds on the distribution of DNA tracks. The distributions of DNA tracks for constant (*σ*_*v*_ = 0, blue) and variable (*σ*_*v*_ ≠ 0, orange) replication forks’ speeds at three different time points within the S phase: 30, 45, and 60 minutes. The differences in DNA track distributions between constant and variable forks’ speed models become most pronounced at at later times. The dashed red line denotes the mean value.

First we will illustrate the impact of variance in forks’ speeds on the distribution of the DNA tracks by analyzing single origin of replication. In Fig 2 we show the distribution of DNA tracks (Δ*x*) for constant (*σ*_*v*_ = 0) and variable (*σ*_*v*_ ≠ 0) forks’ speeds and for single origin. The difference between variable and constant forks’ speeds are especially pronounced later in the S phase, while the average length of DNA track remains similar for both models. We have shown (Methods Eq 6) that $\sigma_{\Delta x}^{2}$ can be approximated by

$$\begin{array}{c} \sigma_{\Delta x}^{2}=\mu_{v}^{2}\sigma_{t}^{2}+\mu_{\Delta t}^{2}\sigma_{v}^{2}, \end{array}$$ (3)

which implies that stochasticity of distribution of DNA tracks (*σ*_Δ*x*_) not only depends on the average forks’ speed (*μ*_*v*_) and average firing time (*μ*_Δ*t*_) but also on their degree of randomness (*σ*_*v*_ and *σ*_*t*_). On the other hand, considering Eq 3, assuming constant forks’ speed (*σ*_*v*_ = 0), the second term ($\mu_{\Delta t}^{2}\sigma_{v}^{2}$) vanishes. Therefore, fitting *σ*_Δ*x*_ using constant forks’ speed models will lead to over estimation of *σ*_*t*_*μ*_*v*_. Since the average forks’ speed is relatively easy to determine experimentally, fitting distribution of DNA tracks using constant forks’ speed models (*σ*_*v*_ = 0) will result in artificially increased stochasticity of origin firing (*σ*_*t*_), manifesting itself e.g. by known late origins to fire early in S phase, as if they were early origins, as in previous models [23]. We discuss stochasticity of origin firing time in more detail elsewhere (Yousefi et al., in preparation).

### Examining forks’ speeds stochasticity in wild-type yeast cells

**Fig 3:**
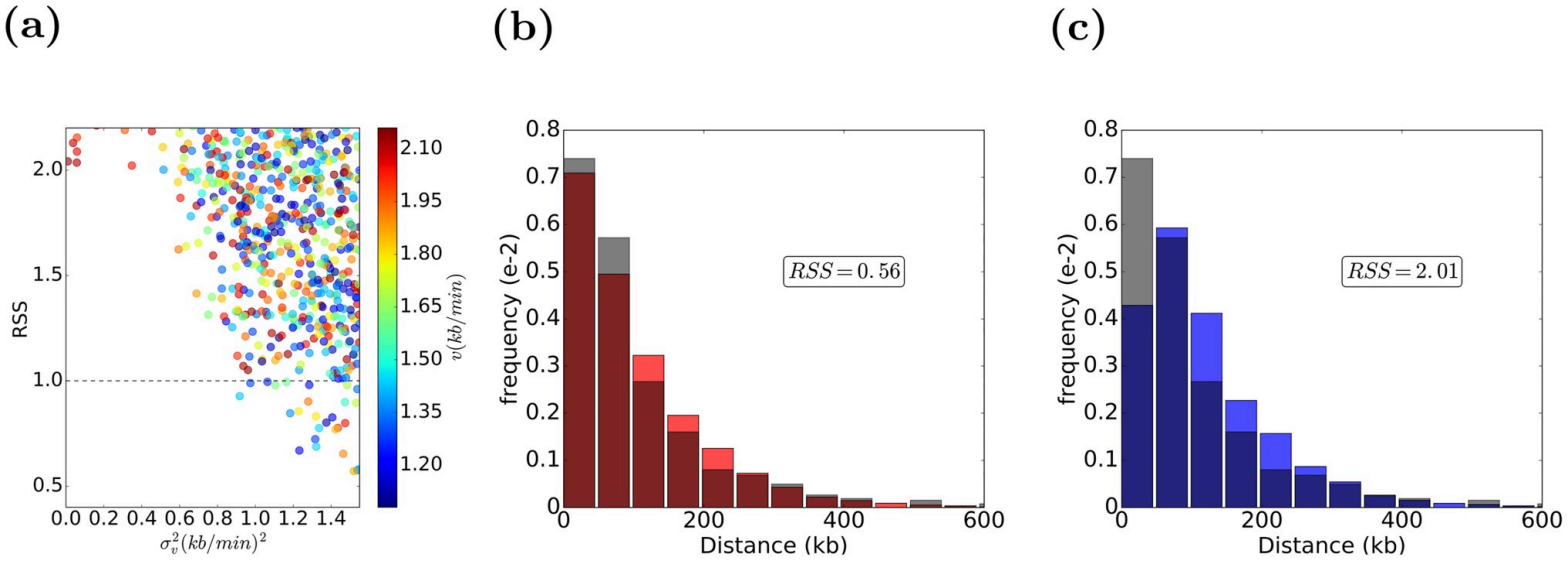
Model selection by Repli-Sim for models with constant and stochastic forks’ speeds. (a) Some models (parameter sets) considered. Stochasticity of replication forks’ speeds ($\sigma_{v}^{2}$) is shown on the horizontal axis, residual sum of squares (RSS) with experimental data (the lower the better) is shown on the vertical axis, the average forks’ speed (*v*) is color coded, as shown in the color-bar. Best models (smallest RSS value) are more stochastic. The best selected constant speed model had parameters ($\sigma_{t}^{2}=5.7min^{2}$, *t*_*exp*_ = 50*min*, *v* = 1.4*kb*/*min*, $\sigma_{v}^{2}=0{\left(kb/min\right)}^{2}$) and the best variable speed model was ($\sigma_{t}^{2}=9.7min^{2}$, *t*_*exp*_ = 42*min*, *v* = 1.5*kb*/*min*, $\sigma_{v}^{2}=0.9{\left(kb/min\right)}^{2}$). The *t*_*exp*_ and fork speed from experimental data are 40*min* and *v* = 1.6(*kb*/*min*), which are more compatible with the variable fork progression model. (b, c) The distribution of DNA tracks for both best stochastic (b, orange) and constant (c, blue) forks’ speeds models are shown along with the distribution of DNA tracks from experimental data (gray), which shows a better fit for the stochastic forks’ speeds model. The average distance traveled in the stochastic forks’ speeds model is compatible with the experimental data (∼105*kb*).

To investigate whether the forks’ speeds are stochastic or constant in wild-type (wt) yeast cells, we used time course DNA copy number sequencing data [23]. This experimental data was taken every 5 minutes between minute 15th to 40th during the S phase and included mean firing times and efficiencies of the origins, which we utilized in our analysis. Other parameters including *σ*_*t*_, *σ*_*v*_, *μ*_*v*_, and time of observation *t*_*exp*_ were selected by Repli-Sim through identifying best-fitting model via simulations. For both constant and stochastic forks’ speeds models, the simulations were performed for >5000 different sets of parameters selected by a genetic algorithm; for each parameter set the distribution of DNA tracks, binned with bin size 1*kb*, were derived and compared with the distribution of DNA tracks at 40*min* generated from experimental data using residual sum of squares (RSS), as shown in Fig 3(a). Fig 3(b) and 3(c) present the results for best fitting parameters for both constant and stochastic forks’ speeds and shows that a model with the stochastic forks’ speeds fits the experimental data best. The best fitting model exhibits considerable relative stochasticity of forks’ speeds ($\sigma_{v}^{2}=0.4{\left(kb/min\right)}^{2}$). Strikingly, that same relative stochasticity of replication forks’ speeds that we derived from simulations was observed in in vitro studies of individual replication forks in another organism [11]. Moreover, the average replicated distance is comparable in stochastic forks’ speed model with that of the experimental data (105*kb*). In addition, the average replication forks’ speeds and *t*_*exp*_ derived from simulations for the variable forks’ speeds model (1.5 (kb/min), 42 (min)) are more consistent with experimental data (1.6(*kb*/*min*), 42(*min*)) than those obtained from best constant speed model (1.4(*kb*/*min*), 50(*min*)).

### Examining forks’ speeds stochasticity in hydroxyurea-treated wt yeast cells

**Fig 4:**
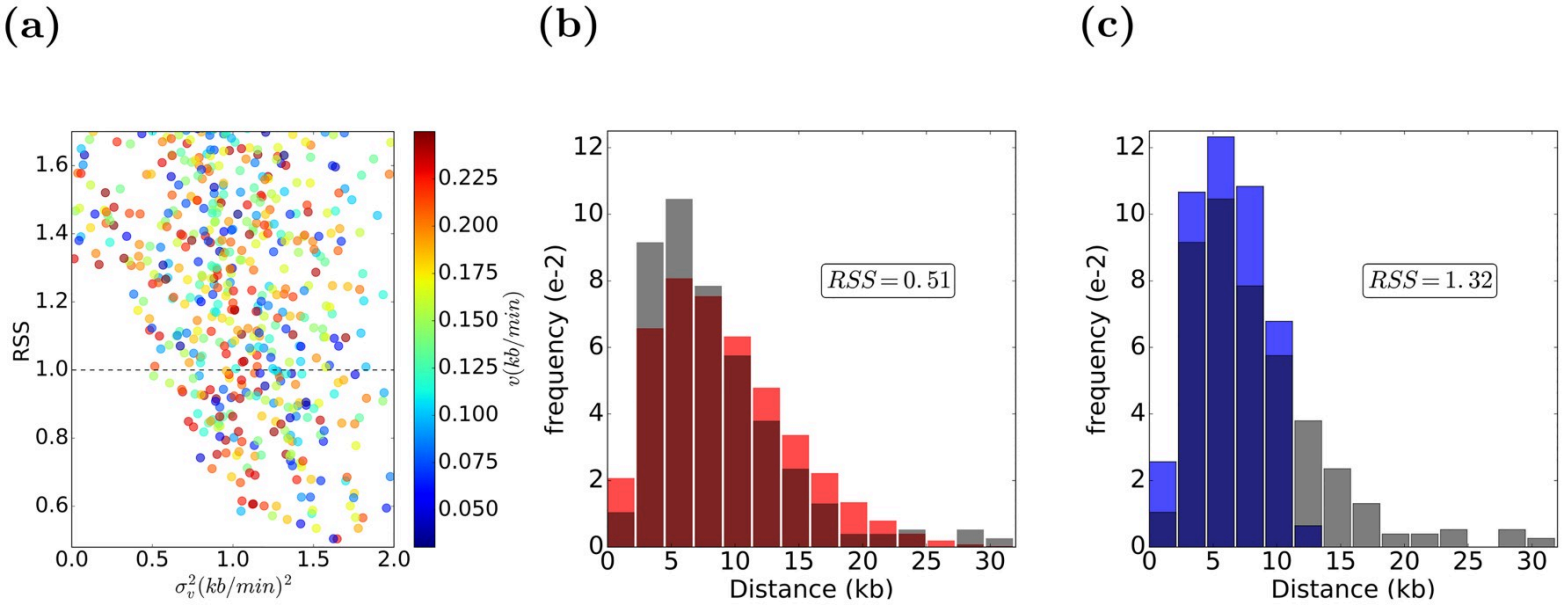
Results of Repli-Sim for HU-treated cells for both constant and variable fork speed models. (a) Some models (parameter sets) considered. Stochasticity of replication forks’ speeds ($\sigma_{v}^{2}$) is shown on the horizontal axis and residual sum of squares (RSS) with the experimental data of [31] is shown on the vertical axis, the average forks’ speed (*v*) is color-coded (side bar). Best fitting models (smallest RSS) are characterized by more stochastic forks’ speeds. Best fitting constant speed model is ($\sigma_{t}^{2}=11\left(min^{2}\right)$, *t*_*exp*_ = 52(*min*), *v* = 0.07(*kb*/*min*), $\sigma_{v}^{2}=0{\left(kb/min\right)}^{2}$,) the best selected variable speed model is ($\sigma_{t}^{2}=7.7\left(min^{2}\right)$, *t*_*exp*_ = 52(*min*), *v* = 0.12(*kb*/*min*), $\sigma_{v}^{2}=0.07{\left(kb/min\right)}^{2}$, *p*_*end*_ = 2*e* − 6). (b, c) The distribution of DNA tracks for both best stochastic (b, orange) and constant (c, blue) forks’ speeds models are shown along with the distribution of DNA tracks from experimental data (gray), which shows a better fit for the stochastic forks’ speeds model.

Hydroxyurea (HU) is an inhibitor of the ribonucleotide reductase (RNR), an essential enzyme for catalyzing the production of deoxyribonucleotide triphosphates (dNTPs), the building blocks of DNA. As a result, HU treatment depletes dNTPs thus slowing replication fork progression and making HU-treated cells an interesting case to study. To examine the stochasticity of forks’ speeds and its impacts on replication in HU-treated cells, we used experimental DNA tracks data from HU-treated wt yeast cells studied in [31]. Mean origin firing time for each individual origin was derived as described in Methods. Similar to the previous analysis, for both constant and stochastic forks’ speeds models, the simulation was run over 5000 sets of different parameters selected randomly by a genetic algorithm, and for each parameter set the distributions of DNA tracks were derived and compared with the experimental DNA track distribution by calculating the RSS between the distributions binned with 1*kb* bin size. We first identified a group of best fitting models (RSS <0.65), and then as the final model we selected the model with a total number of active origins consistent with that of the experimental data (280 ± 10). It is important to note that stochasticity of firing time *σ*_*t*_ impacts origin usage. A smaller *σ*_*t*_ is equivalent to more localized firing time, which leads to activation of fewer late origins early in S phase as compared to a larger *σ*_*t*_, as we discuss elsewhere (Yousefi et al., in preparation). Indeed, the dysregulation of origin activation has been observed in various conditions [31–34], which could be explained by increasing stochasticity of firing time of origins of replication *σ*_*t*_. Some parameter sets from simulations are presented in Fig 4(a), where models with a smaller RSS value (i.e. better fitting), exhibit more stochasticity in fork speed (higher *σ*_*v*_). The best models are selected using numbers of active replication origins, as described above. In Fig 4(b) and 4(c) we compare experimental and best-fitting simulated distributions of DNA tracks for constant speed and stochastic forks’ speeds models. For untreated wt yeast cells stochastic forks’ speeds model fits the data much better.

#### Parameter selection

For parameter selection, a genetic algorithm is used for minimization of the RSS between the distribution of DNA tracks from experimental data and simulation results. We used a population of 5000 sets of parameters, run in parallel using the open source implementation OpenMP over 32 threads. For each condition, a number of best sets of parameters is selected, as shown in Figs 3 and 4, among which the one with the total number of active origins most similar to the experimental data is chosen.

### Comparison with the previous work

**Fig 5:**
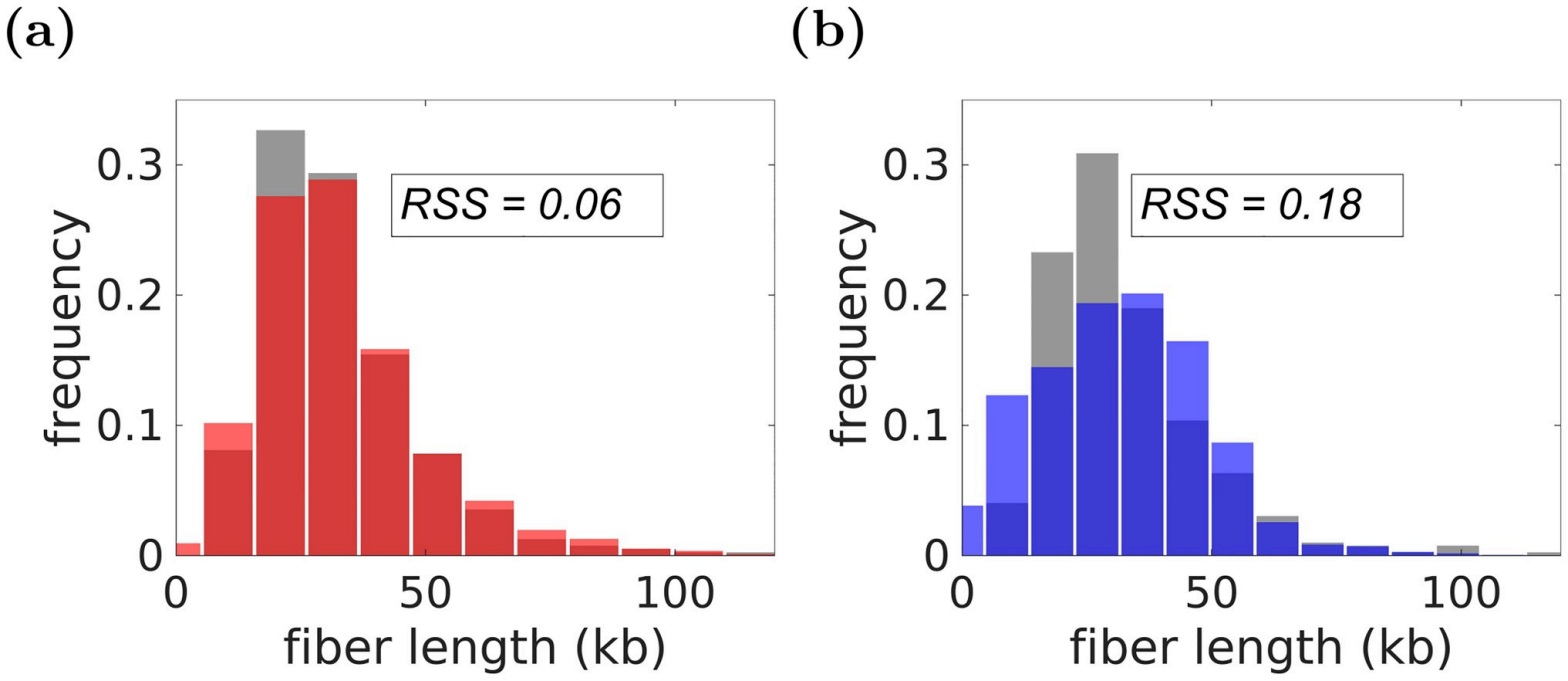
Comparison of DNA track distributions predicted by different models with experimental data. (a) Our model (random origin activation times, but stochastic forks’ speeds), (b) Hawkins et al. model. Our simplified model (a, orange) reproduces distribution of distances travelled by replication forks measured by DNA combing (gray) much better than previous model (b, blue).

To compare Repli-Sim fairly with the most current published model of DNA replication [23], we obtained new DNA track data from untreated wild-type yeast cells for which the Hawkins et al. model [23] was optimized. In the Hawkins et al. model, origins have not only individual assigned firing time, $\mu_{t}^{i}$, but unlike in our model, each origin has its individual firing speed stochasticity, $\sigma_{t}^{i}$, resulting in 814 model parameters. Since the Hawkins et al. model was developed for wild-type yeast used in this comparison we retained their origin parameters. The fork speed for Hawkins et al. model was optimized to maximize fit with the data. We calculated Kolmogorov-Smirnov statistic (maximal distance between the cumulants) for each model and experimental data and selected the model with minimal value of this statistics (Fig 3(b) and 3(c)). To stress the impact of stochasticity of replication forks’ speeds on the accuracy of reconstruction of DNA replication dynamics, we prepared a simplified Repli-Sim model, where origin firing time will be stochastic (not empirically derived as previously). Such a simplified Repli-Sim model only has 5 parameters, in addition to origin coordinates, considered known. Again, Kolmogorov-Smirnov statistics was used to select the Repli-Sim model best fitting experimental data. As Fig 5 shows, even such a simplified Repli-Sim model fits the DNA tracks data much better than much more complex Hawkins et al. model [23]. This result highlights the key role stochasticity of replication forks’ speeds plays in accurately reconstructing dynamics of DNA replication and thus DNA tracks.

### Context-dependent variability of fork speed and its impact on the completion of replication

**Fig 6:**
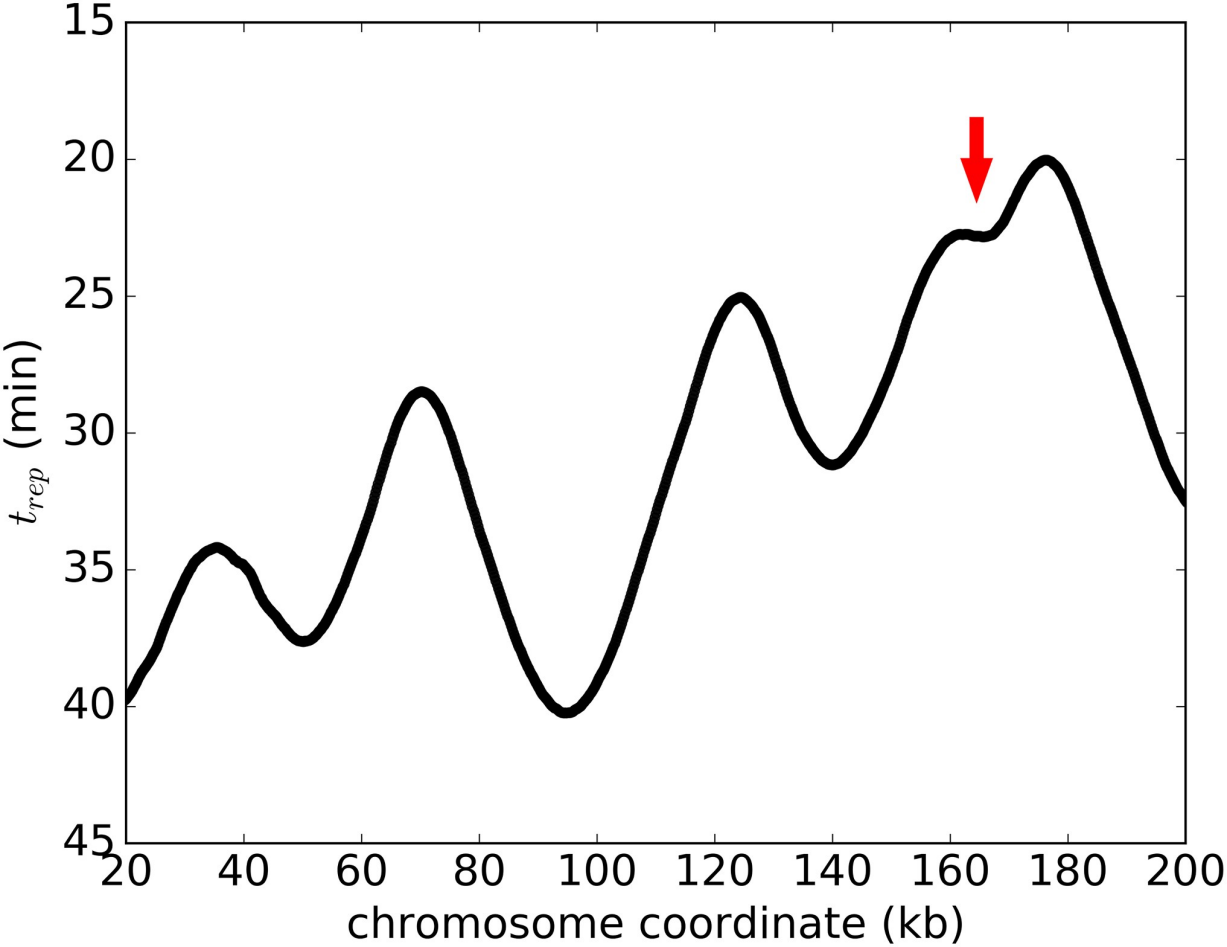
Replication profile derived from our best model: Stochastic forks’ speed not depending on genomic location and stochastic origin firing (*σ*_*t*_ ≠ 0). Note that the replication profile slope is highly variable (as indicated by the red arrow) even though the fork speed is constant. Here, the variation in slope is due to the origin firing with different probabilities at different times, although such variation can be also caused by local variability of forks’ speeds.

DNA replication dynamics is impacted not only by origin activation, but also by replication forks’ speeds. The replication profile shows time at which 50% of the DNA was replicated in a given genomic location (Fig 6). It has been proposed in [12, 13, 35] that the slope of the replication profile curve between successive minimums and maximums can be interpreted as the average forks’ speed in that region. However, such assumption is not valid because of the complexity of replication profile, which is affected by many parameters including temporal profile of the origins activation and speeds of the forks emanating from them. This point is illustrated in Fig 6, where we simulated such a curve based on stochastically variable forks’ speed and experimentally derived intervals of origin firing. Even though the forks’ speed does not depend on the genomic location, the slope of the curve, proposed to be proportional to the forks’ speed, changes substantially between genomic regions. Fig 6 shows that deriving the average forks’ speed from the slope of the replication profile may lead to inaccurate estimation of forks’ speed progression as in [12] and incorrect conclusion that the average forks’ speed must be changing depending on the genomic regions [35][12].

**Fig 7:**
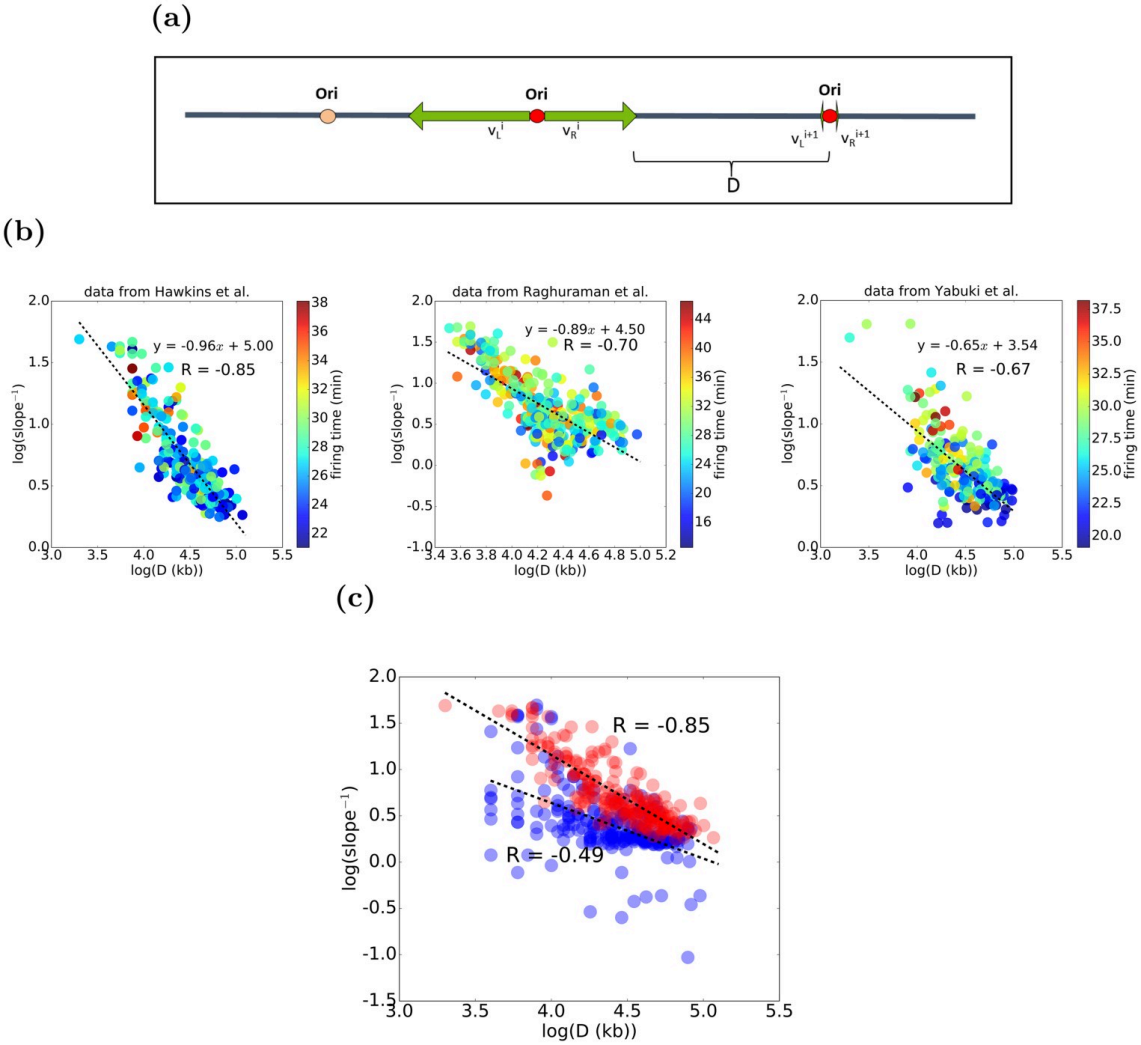
Replication fork speed adjustment based on the distance to the approaching fork. (a) Schematic representation of *D*, the distance from an emerging fork to an approaching fork. (b) The strong correlation between *D* and emerging fork speed is observed in three independent data sets [12, 13, 23] (c) dependence of the slope on the inter-origin distance observed in the experimental data (red) cannot be reproduced by our model where forks’ speed varies stochastically but does not depend on the genomic location (blue).

On the other hand, it is true that local change in forks’ speed would impact the slope of the replication profile curve. Therefore, we analyzed local slopes of the replication profile curve and observed an interesting dependence between the slope of the replication profile curve and the average distance of forks starting from a given origin to the approaching forks (Fig 7(a)). We observed this high correlation (Fig 7(b)) in three independent data sets ([23], [13] and [12]). Moreover, this correlation is not fully explained by our current model, where forks’ speed is stochastic between forks, but does not depend on a genomic region (Fig 7(c)). Motivated by this observation, we implemented an increase in the forks’ speed based on its distance to an approaching fork, while mean forks’ speed remains constant and is consistent with experimental data. To keep model realistic, maximal such an increase in speed is capped at 1.9 average fork speed.

**Fig 8:**
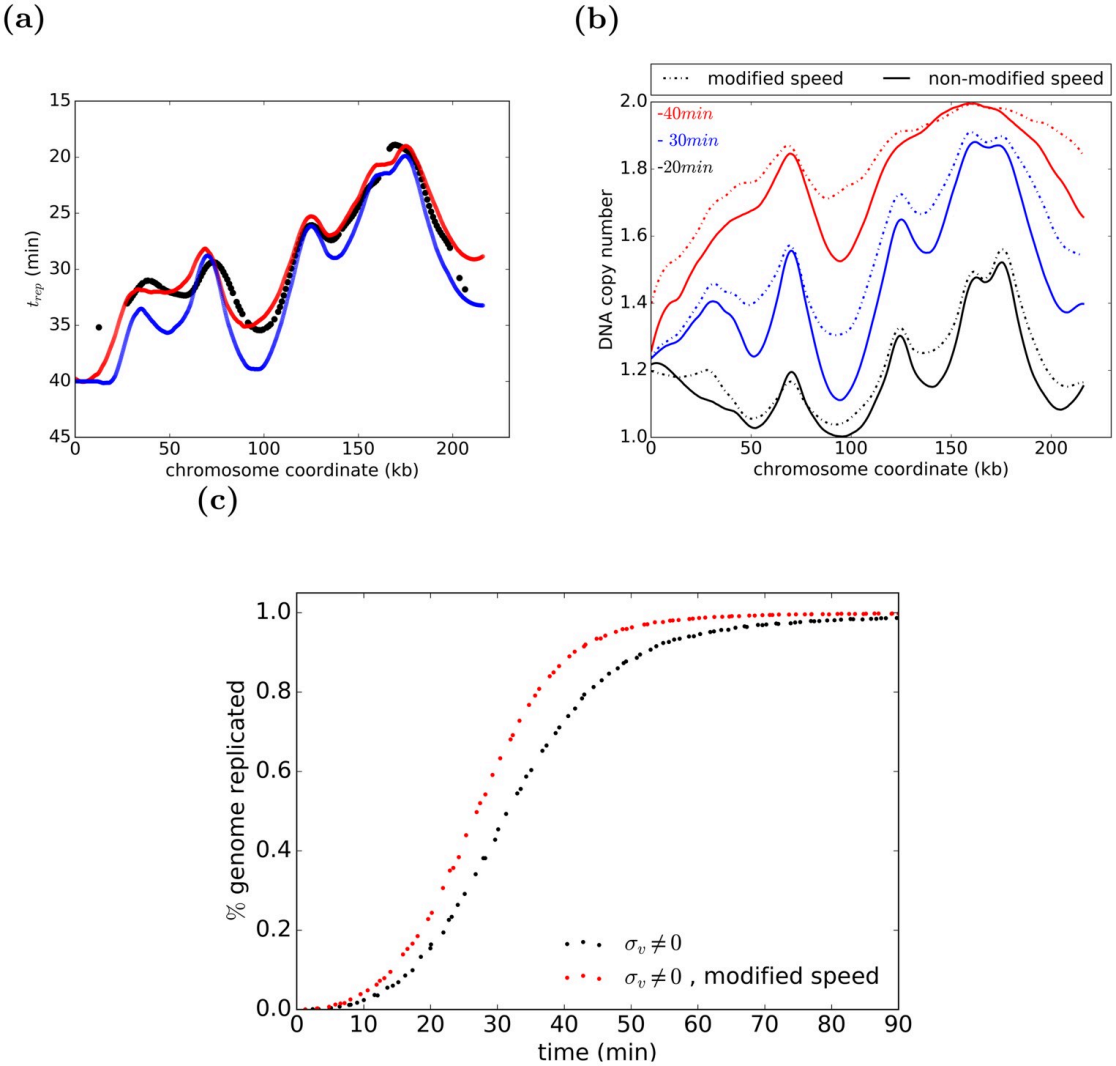
Replication timing profile and DNA copy number of chromosome I and percentage of genome replicated as a function of time. (a) Schematic representation of replication timing profile for experimental data ([23]) (black), along with modified (red) and non-modified (blue) fork speed models, which shows a better fit of the experimental data with modified fork speed model. (b) DNA copy number at different time points for both modified and non-modified speed models. (c) The impact of the fork speed modifications on the dynamics and completion of replication. The percent of replicated genome is presented as a function of time for stochastic forks’ speeds models with regular (black) speed and speed modified based on the distance to approaching fork (red). The modified forks’ speeds model promotes timely completion of the replication.

To examine the impact of the observed increase in the fork speed on the completion of the replication, we compared the experimental data for replication timing profile (the time at which 50% of the DNA at specific coordinates is replicated) of chromosome I [23] with replication timing profile of both thus modified and non-modified stochastic forks’ speeds models. As shown in Fig 8(a), the modified stochastic forks’ speeds model fits the experimental data the best. The mechanistic explanation for this increase in forks’ speed can be a synergy effect for unfolding of DNA in front of a replication fork, resulting in faster fork progression. This plasticity of the forks’ speed could be the reason for the higher stochasticity of DNA track distribution observed for later firing origins [24].

The stochastic nature of replication leads to the “random replication gap problem” [36–40]. Namely, random origin firing will occasionally lead to large gaps between replication bubbles that would take a very long time to replicate. Such gaps challenge the completion of DNA replication in a timely manner. All the proposed solutions to address this problem has been focusing on the regulation of origin activation [37, 38], while regulation of replication fork progression, which impacts the dynamics of replication as well, has not been taken into account. Specifically, it has been proposed [37] that probability of activation of replication origins increases as S phase progresses and therefore origins located in yet-unreplicated gaps have the higher chances to be replicated the longer the gap persists thus promoting timely completion of the replication. In contrast, based on our data analysis, we propose that the forks approaching each other accelerate their speed, perhaps due to cooperative chromatin unwinding, and thus promote completion of the replication. Our solution is more general since it is also addressing a situation when no origins are present in the potential gap regions. As shown in Fig 8(b) on the example of DNA copy number profiles for chromosome I for three different time points (20, 30, and 40 minutes), the gaps in replicated DNA are larger in the model with non-modified speed. Indeed, as shown in Fig 8(c), our modified fork speed model promotes the completion of replication, e.g. in the modified stochastic forks’ speeds model 98% of the genome is replicated after 55 mins, consistent with experimental observations, while in non-modified stochastic forks’ speeds model it takes much longer, 79 minutes. Thus the fork speed modification we proposed addresses the random gap problem by promoting timely completion of replication.

### Experimental data

Throughout the analysis, three different experimental data sets ([12, 13, 23]) are utilized with the list of origins of replication detected in each individual experiment. The origins used are consistent with the OriDB database [5]. The DNA fiber data for wt cells collected during S phase, kindly provided by Philippe Pasero, were used for model selection.

### Code availability

All software used in this project is available from https://github.com/rowickalab/RepliSim.

## Discussion

### Key role of stochasticity of replication forks’ speeds

Even though the experimental data, both from single-cell biophysical studies of replication fork and from visualizing DNA track in vivo (DNA combing) indicate that replication forks’ speeds are highly stochastic, all published DNA replication models assumed constant replication forks’ speeds. Here, we presented Repli-Sim, the first model of DNA replication including stochastic replication forks’ speeds. We have shown that Repli-Sim matches DNA tracks travelled by individual forks much better than models employing constant forks’ speed. To illustrate how important stochastic forks’ speeds are for accurate DNA track matching, we simplified our model to only 5 parameters and nevertheless obtained better fit with DNA track data than the much more complex constant forks’ speed model [23], utilizing 814 parameters.

We have shown that standard deviation of the length of DNA tracks (i.e. distances travelled by individual forks), *σ*_Δ*x*_, for each origin can be approximated by the formula:

$$\sigma_{\Delta x}^{2}=\mu_{v}^{2}\sigma_{t}^{2}+\mu_{\Delta t}^{2}\sigma_{v}^{2},$$

where *μ*_Δ*t*_ is the average time elapsed since the origin was activated. This formula shows why the previous modeling attempts, assuming a constant replication forks’ speed (*σ*_*v*_ = 0), were not successful: *σ*_*v*_ is an important contributor to *σ*_Δ*x*_, so assuming empirically incorrect *σ*_*v*_ = 0 may force compensation with too large *σ*_*t*_, resulting in incorrectly derived timing of the origin firing. Specifically, too large stochasticity of firing time *σ*_*t*_ leads to activation of many origins in early S phase, including origins typically firing only in late S phase, as in [23]. Alternatively, if stochasticity of firing time *σ*_*t*_ has correct value, and *σ*_*v*_ is assumed zero, it leads to substantially distorted DNA tracks distribution *σ*_Δ*x*_. On the other hand, *μ*_Δ*x*_ is not affected by stochasticity of forks’ speeds, which is why the stochasticity of forks’ speeds is most apparent in data on distances travelled by individual replication forks, such as DNA tracks, where *σ*_Δ*x*_ becomes visible.

### Model selection

During model development, a better fit can normally be achieved with an increased number of parameters, which may lead to overly complicated models and over-fitting (learning noise). To avoid over-fitting, we assume the same *σ*_*t*_ for all origins and do not attempt to match the data to individual origins. Nevertheless, our modeling results also in a good fit near origins, as we show in (Yousefi et al., in preparation). Moreover, using single *σ*_*t*_ instead of >400 individual *σ*_*t*_(*i*) as in [23], gives clearer insights into changes in replication program in HU-treated cells, as we discuss elsewhere (Yousefi et al., in preparation). For the same reasons, we do not consider potential changes in replication forks’ speeds depending on genomic regions.

### Model modification promoting timely completion of replication

Our basic model assumes that forks’ speeds are stochastic but that the speed of each individual fork is constant. We also proposed a modification where the speed of forks activated later in replication can be increased up to 1.9 average speed depending on its distance to the approaching fork. Such modification promotes the timely completion of replication by addressing the “random replication gap problem”, discussed above. We hypothesize that observed apparent speed up of a replication fork when approaching fork is present nearby is caused by the dependence of fork speed on topology of the DNA molecule. If such dependence indeed exists it should manifest itself also in other situations. Dependence of the fork speed on topology of the DNA molecule can be added to our model, if desired. We did not currently implement it both to avoid complicating the model (our current model accurately reproduces the test data) and due to lack of appropriate training and input data. Other modifications, e.g. largely increasing average speed of replication, may have a similar effect on facilitating completion of replication, but our modification has the advantage of utilizing the average replication speed consistent with observations (1.6 kb/min).

### Model limitations

It is known that replication forks stall on natural Replication Fork Barriers and non B-DNA structures [41]. Not including variability of fork speed potentially related to these impediments is a limitation of our current model, which can however be addressed within the framework we proposed. We chose to currently not to implement locally variable fork speed for several reasons. First, there is lack of experimental evidence that local variance in fork speed is substantial. As we discussed (Fig 6) a naive interpretation of replication profile curves may lead to impression that local variability of replication fork speed is prevalent and high. However, our simulations show that observed replication profile curves are highly consistent with fork speed not depending on genomic coordinates (Fig 8(a)). Second, implementing locally variable fork speed will add hundreds, if not thousands, parameters. These parameters will be difficult to fit correctly. Therefore, we are convinced that either our simpler, but accurate, model should be used or fork speed modification depending only on distance to the approaching fork (single parameter) should be implemented.

### Applications and future directions

DNA replication is a complex process, with elaborate spatio-temporal regulation, which remains incompletely understood. Due to this complex regulation of replication, it is difficult to infer the role individual proteins play in regulation of DNA replication from genomic DNA copy number variation or DNA track data, since it is difficult to distinguish between changes in replication forks’ speeds and origin activation in such data. Here, we presented Repli-Sim, a probabilistic model of DNA replication including stochastic replication speed. We have shown that Repli-Sim accurately reproduces experimental data. Moreover, Repli-Sim allows the user to classify experiments in terms of fundamental parameters of replication, such as average replication forks’ speed, stochasticity of forks’ speeds, and stochasticity of origin firing. Such presentation allows us to better understand the impact that individual treatments and proteins have on DNA replication, as well as compare conditions in this space of fundamental replication parameters (Yousefi et al., in preparation). Another application of our simulations can be studying replication stress and DNA double-strand breaks (DSBs) originating from broken replication forks. Currently, the numbers of DSBs per cell can be precisely measured genome-wide using qDSB-Seq [42]. However, since replication stress typically substantially changes the replication program, increased numbers of breaks per cell do not have to mean that forks break more often. Therefore, combination of DNA replication simulation by Repli-Sim with the landscapes of DSBs measured by qDSB-Seq, allows deeper insight into how stalled replication forks break and form DSBs as a result of replication stress [43]. Besides numerous applications of this approach to the general studies of the fundamentals of replication process, analyzing impact of replication forks’ speeds stochasticity could provide better understanding of replication delay induced by ionizing radiations. The distinction between the number of direct DSB and the number of enzymatic DSB resulting from replication fork breakage on other DNA lesions is an important problem in the radiation biology.

Repli-Sim is designed to be general and usable with different input data types, in contrast with [23], which is designed to use with microarray data only. Here, we have shown how Repli-Sim can be used with DNA combing data as an input. Alternatively, Repli-Sim can also use DNA copy number data from sequencing or microarrays as an input, after pre-processing the data to derive DNA track distribution. Such pre-processing has an additional advantage that it acts as a smoothing procedure and reduces the noise. Last but not least, Repli-Sim is very fast, simulations of DNA replication in a given condition require testing of 10,000 sets of parameters, which takes only 7 hours to perform on a 16-core, 32-thread 3.1GHz workstation. Therefore, Repli-Sim can be used to infer spatio-temporal organization of replication in variety of conditions, as long as appropriate data is available. Once Repli-Sim derives parameters of a given state, also spatio-temporal organization of replication and later and earlier time-points can be reconstructed. Therefore, Repli-Sim can play a role similar to the role of high-throughput screening in drug discovery: allowing very fast testing of a research hypothesis using much less data for validation.

Repli-Sim is the first model of DNA replication which allows for stochastic replication forks’ speeds. We have shown that including stochastic replication forks’ speeds is a key innovation allowing correct reconstruction of distances travelled by individual replication forks both in wild-type cells and in a condition when replication stress is induced. We also proposed an empirical modification to the replication fork speed, promoting completion of replication in a timely manner.

## Materials and methods

### DNA replication simulations (Repli-Sim)

Repli-Sim is a probabilistic numerical model designed to study the dynamics of DNA replication. It takes into account two groups of parameters: local and global. Local parameters are individual to each specific origin, while global parameters are those assumed to be approximately similar all across the genome.

During S-phase, origins of replication are activated and DNA tracks (continuous distances covered by replication forks, Fig 1) are formed and elongated throughout the genome until the whole DNA is replicated. In Repli-Sim, coordinates *x* of replication origins are derived from experimental data and filtered using a database of replication origins, OriDB [5]. As shown in Fig 1, two forks are formed and elongate bidirectionally across the genome to form DNA tracks (Δ*x*). For each origin *i* in a cell population, at time *t*_*exp*_ (measured from the beginning of DNA replication), we derive the distribution of Δ*x* based on two assumptions. First, the firing time of the origin, $t_{0}^{i}$, is derived from a normal distribution with a mean firing time $\mu_{t}^{i}$ (specific to that origin and derived from experimental data), and with global standard deviation *σ*_*t*_:

$$t_{0}^{i}∼N\left(\mu_{t}^{i}|\sigma_{t}^{2}\right).$$

Second, individual forks are assigned with different speeds, *v*^*i*^, derived from the same probability distribution with a mean speed, *μ*_*v*_ and standard deviation *σ*_*v*_:

$$v^{i}∼N\left(\mu_{v}|\sigma_{v}^{2}\right).$$

A probability of origin licensing *c*^*i*^ (a priori probability of origin activation) is assigned to each individual origin as a random number between the experimentally measured frequency of that origin activation and 1. Then, a Monte-Carlo method is used to generate activation time $t_{0}^{i}$ for an origin *i* from a Gaussian probability distribution with an experimentally estimated mean activation time $\mu_{t}^{i}$ specific to that origin (below), and a global standard deviation *σ*_*t*_, same for each origin. Individual forks progress with different speeds, constant for each fork, generated using a Monte-Carlo method from a Gaussian probability distribution with a global average speed *μ*_*v*_ and standard deviation *σ*_*v*_.

### Deriving the formula describing $\sigma_{\Delta_{x}}$

Upon origin activation, two forks are formed and elongate bidirectionally across the genome. For each specific origin, two forks replicate a distance of DNA, called DNA tracks (Δ*x*). For an origin in a cell population, during S-phase at time *t*_*exp*_ measured from G1, the distribution of Δ*x* is derived considering the following assumptions:

1. The firing time of the origin, taken as the initial time (*t*_0_), is derived from a normal distribution with a mean firing time *μ*_*t*_, specific to that origin reproducible from experimental data, and standard deviation *σ*_*t*_:
  $$t_{0}∼N\left(\mu_{t}|\sigma_{t}^{2}\right)$$
2. Individual forks have different speeds, however the speed of each fork is derived from the same probability distribution with a mean speed, *μ*_*v*_, equivalent to the average fork speed observed from experimental data, and standard deviation *σ*_*v*_:
  $$v∼N(\mu_{v}|\sigma_{v}^{2})$$

Considering the relation Δ*x* = *v* ⋅ Δ*t*, using the distribution function of Δ*t* and *v*, the distribution function of Δ*x* for each origin can be derived as follows:

$$\begin{array}{r} \Delta t=t_{exp}-t_{0}∼t_{exp}-N\left(\mu_{t}|\sigma_{t}^{2}\right)∼\\ ∼N\left(t_{exp}-\mu_{t}|\sigma_{t}^{2}\right)∼N\left(\mu_{\Delta t}|\sigma_{t}^{2}\right), \end{array}$$

where *μ*_Δ*t*_ = *t*_*exp*_ − *μ*_*t*_.

From the other side, assuming *σ*_*t*_*σ*_*v*_ ≪ *μ*_Δ*t*_*μ*_*v*_ ([44]),

$$\begin{array}{c} v\cdot \Delta t∼N\left(\mu_{v}|\sigma_{v}^{2}\right)\cdot N\left(\mu_{\Delta t}|\sigma_{t}^{2}\right)∼N\left(\mu_{v}\mu_{\Delta t}|\mu_{v}^{2}\sigma_{t}^{2}+\mu_{\Delta t}^{2}\sigma_{v}^{2}\right) \end{array}$$ (4)

Considering Eq (4) and taking into account the assumption $\Delta x∼N(\mu_{\Delta x}|\sigma_{x}^{2})$, we have:

$$\begin{array}{c} \mu_{\Delta x}=\mu_{v}\mu_{\Delta t}=\mu_{v}\left(t_{exp}-\mu_{t_{i}}\right) \end{array}$$ (5)

and

$$\begin{array}{c} \sigma_{\Delta x}^{2}=\mu_{v}^{2}\sigma_{t}^{2}+\mu_{\Delta t}^{2}\sigma_{v}^{2}, \end{array}$$ (6)

which shows that variance in DNA track distribution is dependent on variance in firing time of the origins of replication as well as variance in the forks’ speeds.

### Deriving mean firing time from experimental data

**Fig 9:**
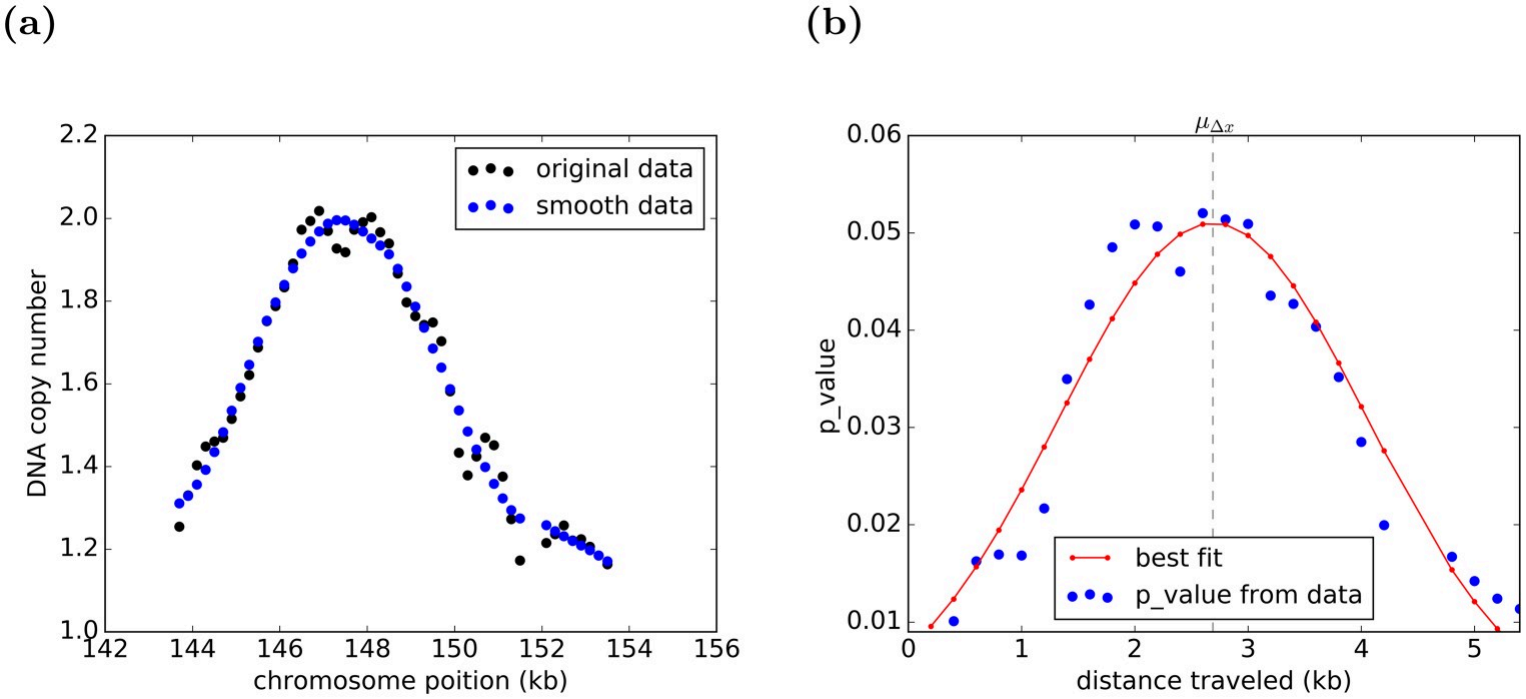
An illustration of the derivation of *μ*_Δ*x*_ for each individual origin. (a) As an example we use the DNA copy number of an early origin located at 147 kb from the beginning of the chromosome I. The distribution of DNA tracks measured from BrdU data [31] is normalized based on the BrdU micro-array DNA copy number of origin *ARS*305, which was verified by quantitative PCR in the same experimental condition. For smoothing the data, the Savitzky-Golary filter (working through the convolution process) is utilized, because it minimally distorts the original data. Maximum of the smoothed peak indicates the origin position. (b) The data are transformed into probability distribution function of Δ*x* and fitted with a Gaussian distribution which peak is assumed to be *μ*_Δ*x*_.

For hydroxyurea-treated wild-type yeast cells, the mean firing time of individual origins is inferred from DNA copy number BrdU-labeled microarray experimental data available in [31]. At each individual origin the distribution of DNA tracks (Δ*x*) is determined and used to derive the mean firing time as follows:

1. To normalize the distribution of DNA tracks measured from BrdU experimental data, the BrdU micro-array DNA copy number of *ARS*305 is used and normalized to give the same efficiency as derived from its DNA copy number from quantitative PCR experiment.
2. The normalized distribution of DNA copy number is used to derive the probability distribution function for each individual origin with a p_value for each DNA track length as shown in Fig 9, from which *μ*_Δ*x*_ is derived.
3. Mean firing time of each origin is assumed to be individual to that origin, however variation of firing time from the mean (*σ*_*t*_) is the same for all the origins and taken as a global parameter. The firing time of *i*^*th*^ origin, (*t*_0_), is derived from the following normal distribution:
  $$t_{0}∼N\left(\mu_{t}^{i}|\sigma_{t}^{2}\right)$$
4. Individual forks have different speeds, however the speed of each fork is derived from the same probability distribution with an average speed *μ*_*v*_ and standard deviation *σ*_*v*_:
  $$v∼N(\mu_{v}|\sigma_{v}^{2})$$

Considering the relation Δ*x* = *v* ⋅ Δ*t*, and taking into account Eq 5, knowing the distribution function of Δ*x*, the mean firing time for each individual origin is derived as follows:

$$\begin{array}{c} \mu_{t_{i}}=t_{exp}-\frac{\mu_{\Delta x}}{\mu_{v}}, \end{array}$$ (7)

which is used in our simulations to infer the mean firing time by implementing *t*_*exp*_ and *μ*_Δ*x*_ while *μ*_*v*_ is the parameter, which is adjusted in the simulation through parameter selection in the genetic algorithm.

The Hill type function was used in the previous work [23] to derive origin firing time. Here, we prefer to assume Gaussian distribution of DNA track lengths Δ*x* because of the good fit with the data, the fundamental nature of the Gaussian distribution and supporting evidence from biophysical studies [11].

## Supporting information

S1 File DNA tracks used in Fig 5. Length of DNA tracks (kb) from HU-treated yeast wt sample collected after 60 min of HU treatment.
(TXT)
